# Parental habituation to human disturbance over time reduces fear of humans in coyote offspring

**DOI:** 10.1002/ece3.4741

**Published:** 2018-12-11

**Authors:** Christopher J. Schell, Julie K. Young, Elizabeth V. Lonsdorf, Rachel M. Santymire, Jill M. Mateo

**Affiliations:** ^1^ Committee on Evolutionary Biology University of Chicago Chicago Illinois; ^2^ School of Interdisciplinary Arts and Sciences University of Washington Tacoma Tacoma Washington; ^3^ USDA‐WS‐NWRC Predator Research Facility, Department of Wildland Resources Utah State University Logan Utah; ^4^ Department of Psychology Franklin and Marshall College Lancaster Pennsylvania; ^5^ Conservation and Science Department Lincoln Park Zoo Chicago Illinois

**Keywords:** *Canis latrans*, hormones, human disturbance, parental effects, repeatability, risk‐taking

## Abstract

A fundamental tenet of maternal effects assumes that maternal variance over time should have discordant consequences for offspring traits across litters. Yet, seldom are parents observed across multiple reproductive bouts, with few studies considering anthropogenic disturbances as an ecological driver of maternal effects. We observed captive coyote (*Canis latrans*) pairs over two successive litters to determine whether among‐litter differences in behavior (i.e., risk‐taking) and hormones (i.e., cortisol and testosterone) corresponded with parental plasticity in habituation. Thus, we explicitly test the hypothesis that accumulating experiences of anthropogenic disturbance reduces parental fear across reproductive bouts, which should have disparate phenotypic consequences for first‐ and second‐litter offspring. To quantify risk‐taking behavior, we used foraging assays from 5–15 weeks of age with a human observer present as a proxy for human disturbance. At 5, 10, and 15 weeks of age, we collected shaved hair to quantify pup hormone levels. We then used a quantitative genetic approach to estimate heritability, repeatability, and between‐trait correlations. We found that parents were riskier (i.e., foraged more frequently) with their second versus first litters, supporting our prediction that parents become increasingly habituated over time. Second‐litter pups were also less risk‐averse than their first‐litter siblings. Heritability for all traits did not differ from zero (0.001–0.018); however, we found moderate support for repeatability in all observed traits (*r* = 0.085–0.421). Lastly, we found evidence of positive phenotypic and cohort correlations among pup traits, implying that cohort identity (i.e., common environment) contributes to the development of phenotypic syndromes in coyote pups. Our results suggest that parental habituation may be an ecological cue for offspring to reduce their fear response, thus emphasizing the role of parental plasticity in shaping their pups’ behavioral and hormonal responses toward humans.

## 1 INTRODUCTION

Maternal effects have the potential to drive both the direction and strength of evolutionary change in a population (Bonduriansky & Day, 2009; Marshall & Uller, 2007; Wolf, Brodie, Cheverud, Moore, & Wade, 1998). A fundamental assumption of maternal effects theory is that parents can vary their phenotype as a result of acute changes to environmental conditions, accrued environmental experiences over time, or both (Badyaev & Uller, 2009; Maestripieri & Mateo, 2009; Mousseau, 1998; Mousseau, Uller, Wapstra, & Badyaev, 2009). Despite the centrality of this assumption, most prior work is commonly conducted within a single reproductive bout or season (Marshall & Uller, 2007). These investigations are seldom representative of lifetime maternal fitness (Badyaev & Uller, 2009; Marshall & Uller, 2007; Plaistow, St. Clair, Grant, & Benton, 2007), particularly because maternal effects in one reproductive season may not be predictive of future reproduction due to fluctuations in predation pressures (Marshall & Keough, 2004; Sheriff, Krebs, & Boonstra, 2010), population densities (Dantzer et al., 2013; Plaistow & Benton, 2009), and resources (Forest, Dender, Pitcher, & Semeniuk, 2017; Hafer, Ebil, Uller, & Pike, 2011; Plaistow et al., 2007). While time is an important component of parental effects theory, particularly because such processes are inextricably linked with a mother's ability to translate temporally‐varying environmental conditions into offspring phenotypes (Uller, 2008), few studies have investigated variation in maternal effects over time (Benson, Mills, Loveless, & Patterson, 2013; Marshall & Keough, 2004; Plaistow et al., 2007; Sheriff et al., 2010).

The extent to which parental effects allow parents to mold offspring phenotypic development has received substantial empirical attention over the past decade (Benard & McCauley, 2008; Champagne, 2008; Champagne & Curley, 2009; Crino, Prather, Driscoll, Good, & Breuner, 2014; Duckworth, Belloni, & Anderson, 2015; Hinde et al., 2014; Kemme, Kaiser, & Sachser, 2007; Love, Mcgowan, & Sheriff, 2013; O'Connor, Norris, Crossin, & Cooke, 2014; Uller, 2008; Weaver et al., 2004). Of primary concern in such studies is whether parental phenotype can act as a reliable cue for offspring to use in maximizing their fitness in the current environment (Uller, 2008; Uller, Nakagawa, & English, 2013). This hypothesis necessarily assumes that parental response is sufficiently plastic to adjust to immediate environmental constraints, and offspring are developmentally plastic enough to match parental response changes (Burgess & Marshall, 2014; Uller, 2008; Uller et al., 2013). Evidence supporting this hypothesis has traditionally investigated how predation regimes (Sheriff et al., 2010; Stein & Bell, 2014), density‐dependence (Dantzer et al., 2013), or resource availability (English, Bateman, Mares, Ozgul, & Clutton‐Brock, 2014; Hafer et al., 2011) induce parental and offspring plasticity. Rarely has the contribution of anthropogenic disturbance been considered (Greenberg & Holekamp, 2017; Miranda, Schielzeth, Sonntag, & Partecke, 2013).

Previous empirical work provides evidence to suggest that wildlife perceive humans as predators, and as such, display fear responses that are qualitatively similar to those exhibited in the presence of natural predators (Blumstein, 2006; Carrete & Tella, 2017; Rebolo‐Ifran et al., 2015). This is particularly the case for carnivores, as several recent studies suggest behavioral and ecological patterns of such species are directly modified as a function of anthropogenic disturbance (Clinchy et al., 2016; Moll et al., 2018; Smith et al., 2017; Smith, Thomas, Levi, Wang, & Wilmers, 2018; Wang, Smith, & Wilmers, 2017). Given that wildlife encounters with humans have gradually become more frequent across the globe within recent decades (Barrett, Stanton, & Benson‐Amram, 2018; Ditchkoff, Saalfeld, & Gibson, 2006), it has become necessary for animals to increase their tolerance of human presence to survive in human‐dominated landscapes (Lowry, Lill, & Wong, 2013; Miranda, 2017; Sol, Lapiedra, & González‐Lagos, 2013). Indeed, recent findings emphasize the significance of human disturbance as a source of ecological variance, suggesting that organisms with frequent human encounters (e.g., urban vs. rural individuals) will show reduced fear of, and habituation to, humans over time (Carrete & Tella, 2013; Cook, Weaver, Hutton, & McGraw, 2017; Martin & Réale, 2008; Uchida, Suzuki, Shimamoto, Yanagawa, & Koizumi, 2016; Vincze et al., 2016). Examining the mechanisms that bolster wildlife habituation to human presence both improves our understanding of the functional significance of human‐mediated behavioral plasticity (Love et al., 2013), and informs us on the processes that may contribute to the development of problematic behaviors linked to human‐wildlife conflict (Blackwell et al., 2016; Soulsbury & White, 2015). Thus, prior research on both wildlife habituation and parental effects provide a framework to explore whether human disturbance over time changes parental cues (i.e., behavior) that offspring use to modify their phenotypes.

**Figure 1:**
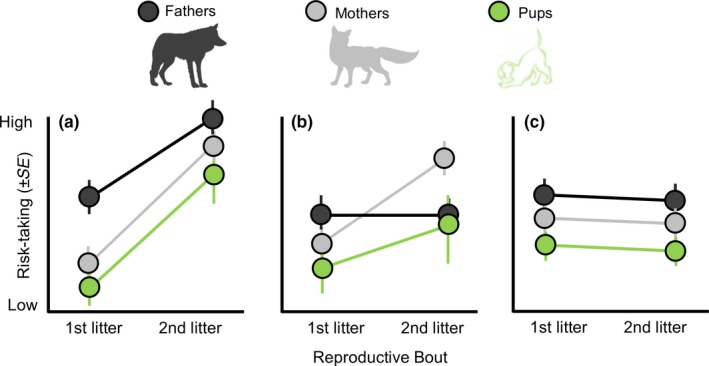
Conceptual diagram of potential scenarios in which parental habituation and offspring risk‐taking behavior are related to predictable cues of anthropogenic environments over time. Dots indicate risk‐taking behavior within fathers (black) and mothers (gray) over successive reproductive events, whereas pups (green) are separate litters. In (a) both mothers and fathers become habituated, and as a result demonstrate riskier behavior across reproductive bouts. If parental cues are a reliable signal of current environmental conditions, then it is predicted that pup risk‐taking will also increase. In (b) only a single parent becomes habituated to anthropogenic disturbance, with the other parent possibly selectively constrained. Second‐litter pups may exhibit slightly greater risk‐taking than their first‐litter siblings, although they may not differ statistically. And in (c), neither parent becomes habituated over time. In all scenarios, it is assumed that parental behavior is a reliable cue of environmental conditions that offspring use to fashion their behavior

In this study, we investigated whether changes to parental fear of humans across reproductive episodes differentially affects fear and endocrine responses of offspring born to separate litters (Figure 1). We test this hypothesis in coyotes (*Canis latrans*), a biparental canid that produces several litters and maintains lifelong monogamous bonds (Hennessy, Dubach, Gehrt, Resources, and Resources (2012)) with near‐equal rates of parental care between mothers and fathers (Schell, Young, Lonsdorf, Mateo, & Santymire, 2018). Our four main questions are as follows: (a) is parental fear of humans reduced over time (i.e., from the first to second reproductive event); (b) does among‐year plasticity in parental fear predict among‐litter plasticity in risk‐taking behavior; (c) do endocrine traits (e.g., cortisol and testosterone) differ between first and second‐litter siblings; and (d) are offspring traits repeatable and heritable? We address these questions in a captive system because the experimental design of among‐litter studies often requires recapture and repeated measures that are difficult to obtain in the wild. Indeed, only two studies prior to this one have observed among‐litter phenotypic plasticity of single mothers (Margulis, Nabong, Alaks, Walsh, & Lacy, 2005; Sheriff et al., 2010), both of which were in captive systems. Moreover, previous evidence suggests behavior in captivity can predict personality variation in the wild (Cole & Quinn, 2014; Herborn et al., 2010), underscoring the ecological significance of such studies.

From our questions, we make several predictions (Figure 1). First, we predicted that parents would exhibit reduced fear responses with their second versus with their first litters. Parents with their second litters have accumulated more experiences of humans than with their first litters, and thus should be more habituated (Figure 1). We quantified risk‐taking behavior as the willingness to forage with persistent human disturbance (i.e., human observer). This is similar to previous studies that assess individual differences in risk‐taking and boldness in relation to anthropogenic disturbance (Dammhahn & Almeling, 2012; De Meester et al., 2018; Greenberg & Holekamp, 2017; Patrick, Charmantier, & Weimerskirch, 2013; Samia, Nakagawa, Nomura, Rangel, & Blumstein, 2015). Second, we predicted that second‐litter pups would be less risk‐averse than their first‐litter siblings. This prediction necessarily assumes that parental habituation can operate as a cue for offspring to modify their behavior accordingly (Figure 1). Third, we predicted that developmental testosterone, but not cortisol, would be lower in second versus first‐litter siblings. Previously, we demonstrated that parents had reduced gestational testosterone (but not cortisol) as experienced versus naïve parents (Schell, Young, Lonsdorf, Mateo, & Santymire, 2016). Hence, our third prediction assumes that parent‐offspring endocrine responses will positively covary as found in previous work (Meylan, Miles, & Clobert, 2012; Sheriff et al., 2010, 2017). Finally, we predicted that risk‐taking would be consistent within individuals (i.e., demonstrate repeatability) as recent literature suggests that fear of humans is highly repeatable and heritable (Carrete & Tella, 2011, 2013; Carrete et al., 2016). To address this prediction, we used a quantitative genetic approach that allowed us to estimate the contribution of additive genetic, permanent environment, maternal, and cohort effects on all pup traits, calculate repeatability, and estimate correlations among pup traits. This study represents a novel integration of parental effects theory with human‐wildlife interactions to assess how human disturbance may contribute to transgenerational plasticity.

## 2 METHODS

### 2.1 Study animals and housing

**Figure 2:**
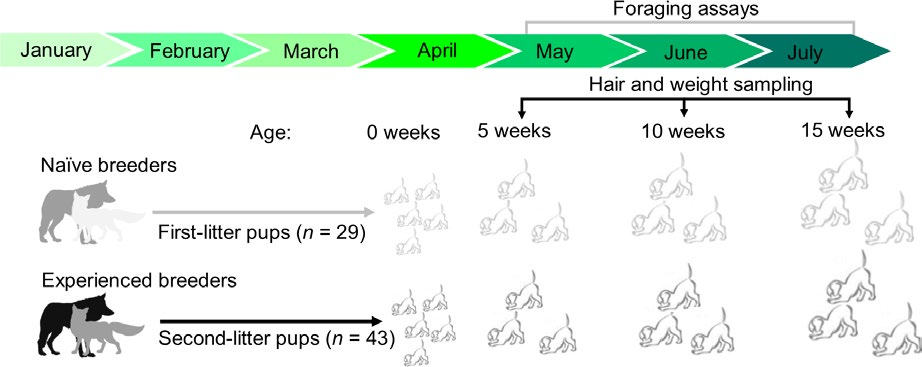
Schematic depicting the general timeline and experimental design used to observe offspring traits of first and second litters. Foraging assays were performed 2–3 times per week from 5–15 weeks of age. At 15 weeks of age, pups were removed from their natal pens to enclosures independent of their parents

We observe a captive coyote population, maintained for research purposes, at the United States Department of Agriculture (USDA) – National Wildlife Research Center's (NWRC) Predator Research Facility, in Millville, UT. Eight breeding pairs (male‐female pairs) and their offspring were observed in 2011 and 2013, and all pairs were nulliparous (i.e., had no prior parenting experience) before the study (Figure 2). In 2011, all parents were less than two years of age (1.4 ± 0.1 years [*X* ± *SD*]). Pups were born in March and April of both years and observed from 5–15 weeks of age. This age was selected because pup emergence from natal dens becomes more frequent, pups are progressively weaned by their mothers, and pups refine their social skills and conspecific communication (Bekoff & Wells, 1982; Fentress, Ryon, & McLeod, 1987; Messier & Barrette, 1982; Sacks & Neale, 2001; Way, Auger, Ortega, & Strauss, 2001). Parent‐pup family units were housed in 1,000‐m^2^ outdoor pens from gestation, in early January, until dispersal age in the wild, in late July or early August (i.e., 15 weeks of age; Bekoff & Wells, 1982). Pups were then relocated from their natal pens to outdoor enclosures separate from their parents to reduce parent‐juvenile conflicts (Figure 2). Outdoor enclosures were equipped with artificial den boxes, multi‐tiered wooden structures for cover, and various small objects for environment enrichment. To reduce the influence of environmental familiarity as a covariate with reproductive bout, parents reared second‐litter offspring in different clover pens than those used during 2011.

### 2.2 Risk‐taking assays

We use modified foraging assays with anthropogenic disturbance (i.e., human observer present) to assess risk‐taking behavior in coyote parents and pups, as seen in previous work (Dammhahn & Almeling, 2012). Although our study coyotes were fed 6 of 7 days weekly by animal care staff leading up to this experiment, our foraging assays varied in two key ways. First, animal care staff scatter feed coyotes; daily food rations are spread throughout a section of their pens instead of placed in specific piles. Second, animal care staff immediately exits the pen and move on to another pen after scatter feeding, so coyotes typically do not eat with a human present unless they start to forage before the staff has completed exiting the pen. Even then, the human is moving and not static. Our design is fundamentally distinct from routine staff procedures in two ways: (a) a single observer intentionally concentrated food in 3–5 piles at the front half of the pen, and (b) that observer then sat at the pen entrance to visually observe focal individuals. We deemed this process as a proxy for human disturbance.

Foraging assays were performed from 5–15 weeks of age, ~2–3 times over the course of each week. We also randomized the order in which pens were observed during each foraging assay. We recorded whether a pup ate at a food pile independent of their parents (i.e., a parent did not bring or regurgitate food to the focal pup) as a binary response (yes/no) over a 7‐min period. Thus, riskier individuals, by definition, ate at food piles more frequently over development than others. We chose a 7‐min observation period because in preliminary feeding observations, this was the maximum amount of time for coyotes within a pen to consume all food provided, regardless of whether few individuals monopolized food rations or if all animals ate.

### 2.3 Pup hormones

Hair has quickly become a viable alternative to quantify individual hormone levels, particularly because hair concentrations represent an accumulated hormonal average over a period of months to years, rather than days (Meyer & Novak, 2012; Schell, Young, Lonsdorf, Mateo, & Santymire, 2017; Stalder & Kirschbaum, 2012). Moreover, recent studies have suggested maternal effects can influence hormone levels in neonatal hair (Dettmer, Rosenberg, Suomi, Meyer, & Novak, 2015; Kapoor, Lubach, Ziegler, & Coe, 2016), are useful in examining developmental patterns in endocrine function (Laudenslager, Jorgensen, & Fairbanks, 2012), show heritable variation (Fairbanks et al., 2011), and are responsive to environmental factors (Salaberger et al., 2016), highlighting the functional significance of hair hormone levels. Consequently, we used hair samples as a means of quantifying repeatable variation in pup cortisol and testosterone over development.

We captured pups at 5, 10, and 15 weeks of age and shaved pups using commercially available pet grooming clippers, which were brushed and wiped with 70% alcohol before each shave. We shaved a 4‐cm area of hair for each individual pup and stored the samples in a plastic bag. Bags were then placed in a drawer to reduce prolonged exposure to direct sunlight, as prior study suggests natural sunlight decreases cortisol concentrations in hair (Wester, van der Wulp, Koper, de Rijke, & van Rossum, 2016). Extraction methodology closely followed (Schell et al., 2017). Briefly, hair was pulverized to a fine powder, combined with 5.0 ml of 90% methanol (methanol:distilled water) and sufficiently agitated over a 5‐hr period. Samples were then dried down and reconstituted with 500 μl of phosphate‐buffered saline solution before running on cortisol and testosterone enzyme immunoassays (EIA). Complete description of EIA methods, including validation and differences as a function of body region, can be found in (Schell et al., 2017). Furthermore, in our previous investigation we did not find any differences in pup cortisol or testosterone concentrations as a function of body region (Schell et al., 2017), as seen in other taxa (Acker, Mastromonaco, & Schulte‐Hostedde, 2018; Carlitz, Kirschbaum, Stalder, & van Schaik, 2014). We were therefore able to compare pup hair samples collected from varying body regions in the current study.

### 2.4 Statistical analyses

We first investigated whether parental risk‐taking behavior changed from the first to the second reproductive bout using univariate generalized Bayesian animal models (i.e., generalized mixed‐models) with Markov chain Monte Carlo (MCMC) estimation (de Villemereuil, 2012; Hadfield, 2010; Wilson et al., 2010). We included litter year (i.e., first vs. second litter), pup developmental age, litter size, and sex as fixed effects in our model. In addition, male‐female pairs in this study were previously exposed to olfactory attractants to simulate high‐density conspecific environments meant to increase glucocorticoid concentrations prepartum (Schell et al., 2016), comparable with prior studies of vertebrate maternal effects (Dantzer et al., 2013; Schweitzer, Schwabl, Baran, & Adkins‐Regan, 2014). Briefly, experimental groups (2011: *n* = 4; 2013: *n* = 4) received the odor cues four times over a 20‐day period, whereas control pairs (2011: *n* = 4; 2013: *n* = 4) received water as a delivery control (Schell et al., 2016). Our initial study did not have an outgroup odor; however, previous studies indicate that coyote behavioral responses toward other chemical attractants are characteristically similar to the behavioral responses we observed in our previous work (Kimball, Johnston, Mason, Zemlicka, & Blom, 2000; Kimball, Mason, Blom, Johnston, & Zemlicka, 2000; Schell et al., 2016; Shivik, Wilson, & Gilbert‐Norton, 2011). We did not find a statistical effect of our odor manipulation on subsequent parenting behavior (Schell et al., 2018) or prolonged hormonal effects (Schell et al., 2016). Nevertheless, we also included parental odor treatment (i.e., “odor”) as a fixed effect in our statistical analyses. All parental models included animal identity (*V_A_, *identity link to the pedigree), individual identity (*V_PE_*, identity), maternal identity (*V_M_*, mother ID), and cohort identity (*V_C_*; common environment or litter) as random effects (Wilson et al., 2010). Because the response variable for risk‐taking behavior was binary, we used a categorical error structure fitted in MCMCglmm with a parameter expanded prior (*V* = 1, *μ* = 1,000, *α.μ* = 0, *α.V* = 1) for the *G* priors (random effects) and the residual variance fixed to one (*V* = 1, fix =1) for the *R* priors, similar to previous studies (Araya‐Ajoy & Dingemanse, 2017; Patrick & Weimerskirch, 2015; Patrick et al., 2013).

To determine whether offspring traits differed as a function of parental reproductive bout, we used univariate animal models with litter (i.e., first or second) included as a fixed effect, as well as all other fixed effect variables found in parental models (i.e., developmental age, sex, litter size, and parental odor treatment). Pup risk‐taking was fit with a categorical error structure with residual variance fixed to 1, comparable with models for parental risk. Endocrine variables, in contrast, were fit with a Gaussian distribution after confirmation of normality using Levene tests implemented from the “Rcmdr” R package (Fox & Bouchet‐Valat, 2018). Thus, models with a Gaussian error structure were fit with uninformative *G* and *R* priors (*V* = 1, *μ* = 1.002), and output was robust to slight changes to these priors.

To investigate whether pup traits (and parental risk‐taking) were repeatable, as well as determine the relative weight of our variance components on trait repeatability, we combined parental and pup data (i.e., all age classes) into a single analysis to effectively estimate variance components in our sample population. In addition, we used our previous univariate models from the first two aims to estimate quantitative genetic components within each age class (i.e., within pups and adults). The total phenotypic variance (*V_P_*) was partitioned into additive genetic (*V_A_, *identity link to the pedigree), permanent environment (*V_PE_*, identity), maternal (*V_M_*, mother ID), and cohort (*V_C_*; common environment or litter) variance parameters by fitting the model with the random terms of “animal”, “ID”, “dam”, and “Litter ID”. Thus, *V_P_* = *V_A_ *+ *V_PE_* + *V_M_* + *V_C_ *+ *V_R_*, in which *V_R_* accounted for the residual variance (i.e., “units”) in the model (Wilson et al., 2010). We estimated narrow‐sense heritability in risk‐taking behavior as *h* ^2^ = *V_A_*/(*V_P_ *+ π2/3), which included the distribution‐specific variance term (π^2^/3) of a binomial model with a logit link (Hadfield, 2010; Nakagawa & Schielzeth, 2010). Permanent environment effects (*PE* = VPE/*V_P_ *+ π2/3), maternal effects (*m* ^2^ = *V_M_*/*V_P_ *+ π2/3), and cohort effects (*C* = *V_C_*/*V_P_ *+ π2/3) were similarly estimated (Petelle, Martin, & Blumstein, 2015; Taylor et al., 2012; Wilson et al., 2010). We then estimated repeatability as the among‐individual variance (*V_I_* = *V_A_* + *V_PE_* + *V_M_* + *V_C_*) divided by the phenotypic variance, *r* = *V_I_*/(*V_P_* + π2/3). Again, we included the distribution‐specific variance term (π^2^/3) of a binomial model with a logit link function. Variance parameters for pup endocrine traits were estimated similarly without the binomial‐specific variance term.

To determine whether offspring traits were correlated, we used a multivariate animal model that contained all fixed and random effects in previous univariate models to estimate genetic, maternal, cohort, and phenotypic correlations among offspring traits. Before analysis, we binned trait data according to the hair hormone survey window, then proceeded to analyze each window separately. In other words, risk‐taking behavioral data were partitioned into a single row with shaved hair samples collected at 10 and 15 weeks of age, respectively. These periods corresponded with ecologically‐relevant developmental periods established in the literature (5–10 weeks: weaning stage; 11–15 weeks: juvenile stage; Bekoff & Wells, 1986; Fentress et al., 1987). Thus, we evaluated each developmental stage separately, with a single pup having a single row of data in the weaning stage, and a single row in the juvenile stage. As a result, we were unable to evaluate permanent environment correlations due to the lack of repeated data (i.e., rows) for each pup within a developmental stage. The remaining random effects were set with an unstructured (“us”) G‐structure, which allowed a fully factorial variance/covariance matrix between pup phenotypic traits and our fixed effects (Boulton et al., 2015; Petelle, McCoy, Alejandro, Martin, & Blumstein, 2013; Sanderson et al., 2015). Litter and the “trait‐1” term were fitted as fixed effects (Wilson et al., 2010). Risk‐taking behavior was fit with the multinomial distribution (“multinomial2”), whereas endocrine variables were fit with a Gaussian distribution. Subsequent phenotypic covariances (*COV_P_*) from the multivariate model were partitioned into additive genetic (*COV_A_*), maternal (*COV_M_*), cohort (*COV_C_*), and residual (*COV_R_*) covariance components. To calculate correlations, we divided the respective covariance estimate for a pair of traits by the square root of the product of variances. For instance, the genetic correlation among pup risk‐taking and cortisol, where “1” is equal to risk‐taking and “2” is equal to cortisol, was calculated as: *r_A_* = COVA_(1,2)_/√(*V*_A(1)_*V_A(2_)); whereas the maternal correlation was calculated as: *r_R_* = COVM_(1,2)_/√(*V_M_*_(1)_ * *V_M_*_(2)_)) (Boulton, Grimmer, Rosenthal, Walling, & Wilson, 2014; Brommer, Karell, Ahola, & Karstinen, 2014; Dosmann, Brooks, & Mateo, 2015; Taylor et al., 2012). To calculate phenotypic correlations, we divided the sum of all covariances (*COV_P_*_(1,2)_ = *COV_A_*_(1,2)_ *+ COV_M_*_(1,2)_ *+ COV_C_*_(1,2)_ *+ COV_R_*_(1,2)_) by the square root of the sum of all variances (*VAR_P_*_(1,2)_ = (*V_A(1)_* *+ V_M(1)_* *+ V_C(1)_* *+ V*_R_ *_(1)_*)*(*V_A(2)_* *+ V_M(2)_* *+ V_C(2)_* *+ V*_R_ *_(2)_*). Hence, phenotypic correlations among traits were estimated as: *r_P_* = COVP_(1,2)_/√(*VAR_P_*_(1,2)_).

All analyses were performed in R version 3.4.4 (R Core Team, 2017). We used the MCMCglmm package to run all Bayesian animal models (Hadfield, 2010) and plots were constructed using ggplot2 (Wickman, 2009). MCMC chains for our animal models were run for 1,000,000 iterations (“nitt”), with the posterior distribution being sampled every 100 iterations (“thin”) after a burn‐in period of 50,000 iterations (“burnin”). In addition, we checked for proper model mixing by examining the levels of autocorrelation (all model runs were <0.04), the variance component plots, and the effective size (all model runs >4,000 per run; (de Villemereuil, 2012; Hadfield, 2010). All models were fit with a pedigree to allow the population variance to be structured among relatives. Sire, dam, grandparental, and great‐grandparental identity were included in the pedigree (Supporting information Appendix S1: Table S1). Estimates for fixed effects (β), repeatability (*r*), heritability (*h* ^2^), and all correlations (*r_A_*, *r_PE_*, *r_M_*, *r_C_*, and *r_P_*) were derived from animal models as the mode of the posterior distribution with accompanying 95% credibility intervals (low CI, high CI) in parentheses. Bayesian estimates were considered statistically significant when the credibility intervals do not overlap zero (Hadfield & Nakagawa, 2010; Sanderson et al., 2015; Stein & Bell, 2015). Lastly, we found that litters were larger in the second (mean ± *SE*: 5.4 ± 1.5 pups) versus the first reproductive bout (mean ± *SE*: 3.6 ± 1.2 pups). Because of the potential confounding relationship between parental parity and litter size, we compared model fit between null animal models containing all previous fixed effects, and alternative models that additionally included the interaction term between litter year and litter size. We then selected the model with the lowest deviance information criterion (DIC) value, in which the optimal model had a ΔDIC = 0 (Hadfield, 2010; Pooley & Marion, 2018; Spiegelhalter, Best, Carlin, & Van Der Linde, 2002). We used the “model.sel” function from the MuMIn package to determine the best‐fit model (Bartoń, 2018), and results are reported from the final (i.e., ΔDIC = 0) animal model.

## 3 RESULTS

Coyote pairs gave birth to their first litters (*n* = 29 total pups in 8 L) in 2011 and second litters (*n* = 43 total pups in 8 L) in 2013 (Figure 2). In 2011, two litters were removed from the study at 10 weeks of age for NWRC‐related research needs. Four additional pups in 2013 (*n* = 4) died of unknown causes at 6 to 7 weeks of age. Thus, we observed *n* = 72 pups up to 5 weeks of age, *n* = 68 pups up to 10 weeks, and *n* = 60 pups up to 15 weeks for a grand total of *n* = 1763 observations of pups and *n* = 790 observations of parents over a 2‐year span. Model selection results can be found in Supporting information Appendix S1: Table S2 and Table S3. For risk‐taking behavior, none of the alternative univariate models containing the interaction between litter year and size performed better than our null models (Supporting information Appendix S1: Table S2). For endocrine traits, however, mixed models with the interaction between litter year and developmental age significantly outperformed null models (Supporting information Appendix S1: Table S3).

### 3.1 Parental and pup risk‐taking

**Figure 3:**
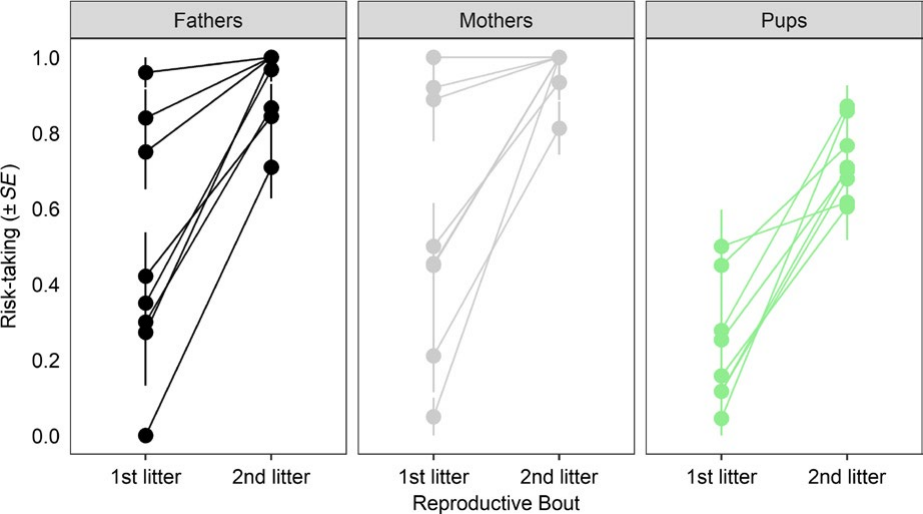
Risk‐taking behavior (i.e., foraging rate) of coyote fathers, mothers, and pups during the first and second reproductive bouts. For mothers and fathers, lines connect the same individuals over time, whereas for pups, lines connect first‐ and second‐litter siblings. Risk‐taking is reported as the average proportion of feeding bouts (±*SE*) in which the individual fed in the presence of a human

Both mothers (mean ± *SE*: 1st year, 0.56 ± 0.12; 2nd year, 0.97 ± 0.02) and fathers (mean ± *SE*: 1st year, 0.48 ± 0.12; 2nd year, 0.92 ± 0.04) were riskier with their second litters than with their first (Figure 3, Table 1a). We did not find evidence of an effect of developmental age, sex, prepartum odor treatment, or litter size on parental risk‐taking (Table 1a). Second‐litter pups had greater risk‐taking compared to their first‐litter siblings (mean ± *SE*, first‐litter pups, 0.16 ± 0.06; second‐litter pups, 0.72 ± 0.04; Figure 3, Table 1b). In addition, pup risk‐taking increased over development (Table 1b). There was no effect of sex, prepartum odor treatment, or litter size on pup risk‐taking (Table 1b). Individual reaction norms for each family unit can be found in Supporting information Appendix S1: Figure S1.

**Table 1:** The influence of litter year (i.e., first vs. second), litter age (in weeks), litter size, sex, and prepartum odor treatment on coyote risk‐taking behavior within coyote (a) adults, (b) pups, and (c) both age classes combined

Fixed effect	(a) β (95% CI)	(b) β (95% CI)	(c) β (95% CI)
Intercept	**−2.398 (−5.862, 1.688)**	**−3.061 (−5.268, −0.950)**	**−2.413 (−4.705, −0.020)**
Litter (1st vs. 2nd)	**3.690 (1.738, 6.046)**	**2.662 (1.580, 4.076)**	**2.705 (1.360, 4.030)**
Age	0.0502 (−0.040, 0.155)	**0.103 (0.063, 0.154)**	**0.094 (0.055, 0.137)**
Litter size	0.632 (−0.107, 1.568)	0.051 (−0.445, 0.570)	0.395 (−0.028, 1.054)
Sex	−0.451 (−2.623, 0.984)	0.028 (−0.425, 0.583)	−0.037 (−0.482, 0.487)
Odor	−0.753 (−2.623, 1.386)	0.517 (−0.647, 1.437)	0.276 (−0.769, 1.395)
Age class	–	–	**−2.273 (−3.664, −1.222)**

Notes

Models (a) and (b) do not examine the fixed effect of age class, as models are partitioned within‐age class. Estimates of fixed effects (β) are given with 95% credible intervals (*n* = 2,553 observations, 89 individuals and 16 cohorts). Estimates that do not overlap zero (i.e., pMCMC < 0.05) are significant and in bold. Null and alternative model comparisons can be found in the Supporting information Appendix S1: Table S2. Final models were chosen based on ΔDIC = 0.

### 3.2 Pup hormones

**Figure 4:**
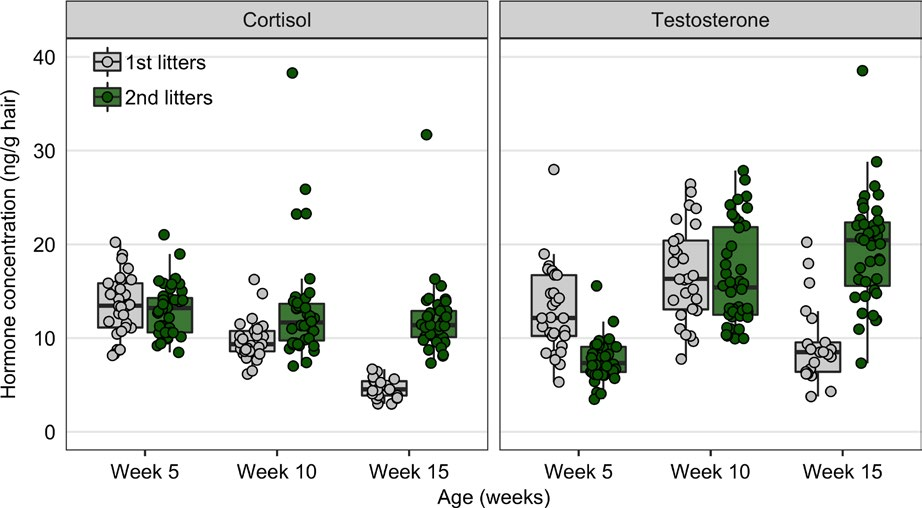
Hormonal differences among first‐ and second‐litter offspring in hair cortisol and testosterone concentrations at 5, 10, and 15 weeks of age. Each dot represents an individual pup

We found that second‐litter pups had higher average cortisol concentrations compared with first‐litter siblings (mean ± *SE*: first‐litter pups, 9.98 ± 0.48; second‐litter pups, 12.73 ± 0.49; Table 2a, Figure 4). Litter year and developmental age were significant predictors of pup cortisol, with a significant interaction between litter and age, suggesting that second‐litter pups had higher cortisol at 10 and 15 weeks of age (Table 2a, Figure 4). Separate‐year litters also differed in their testosterone over development, with a significant interaction term between litter and age (Table 2b). Compared with their first‐litter siblings, second‐litter pups had lower testosterone at 5 weeks of age, but at 15 weeks of age that trend was reversed (Figure 4).

**Table 2:** The influence of litter year, litter age, litter size, sex, prepartum odor treatment, and the interaction term among litter year and age (i.e., litter:age) on coyote pup (a) cortisol and (b) testosterone over development

Fixed effect	(a) β (95% CI)	(b) β (95% CI)
Intercept	**15.678 (12.371, 19.407)**	**16.771 (11.556, 23.224)**
Litter (1st vs. 2nd)	**−5.452 (−8.130, −2.313)**	**−13.418 (−18.530, −8.272)**
Age	**−4.557 (−5.368, −3.385)**	−1.171 (−3.019, 0.571)
Litter size	0.526 (−0.165, 1.270)	−0.395 (−1.414, 0.901)
Sex	0.844 (−0.352, 1.973)	−0.170 (−2.424, 1.584)
Odor	−0.349 (−1.877, 0.876)	0.416 (−2.282, 2.405)
Litter:Age	**3.994 (2.617, 5.130)**	**7.460 (4.931, 9.478)**

Notes

Estimates of fixed effects (β) are given with 95% credible intervals (*n* = 200 hair samples, 72 individuals and 16 cohorts). Estimates that do not overlap zero are significant (i.e., pMCMC<0.05) and in bold. Null and alternative model comparisons can be found in the Supporting information Appendix S1: Table S3. Final models were chosen based on ΔDIC = 0.

### 3.3 Variance component estimates and repeatability

We found evidence of repeatability in risk‐taking behavior across age classes (Table 3), and within each age class (Supporting information Appendix S1: Table S4). We additionally found moderate evidence of maternal and cohort effects on risk‐taking behavior (Table 3). However, we did not find statistical support for additive genetic or permanent environment effects on risk‐taking behavior in coyotes. Both cortisol and testosterone were repeatable (Table 3), despite moderate developmental fluctuations in both hormone traits (Figure 4). Moreover, pup cortisol was mildly heritable, with mild permanent environment, maternal, and cohort effects (Table 3). None of the variance components for testosterone differed from zero (Table 3).

**Table 3:** Heritability (*h^2^*), permanent environmental effects (*PE*), maternal effects (*m^2^*), cohort effects (*C*), and repeatability (*r*) of coyote risk‐taking behavior (both age classes), as well as cortisol and testosterone (pups only)

Trait	*h^2^*	*PE*	*m2*	*C*	*r*
Risk‐taking^a^	0.000 (0.000, 0.167)	0.000 (0.000, 0.127)	**0.171 (0.020, 0.355)**	**0.087 (0.026, 0.256)**	**0.421 (0.281, 0.573)**
Cortisol	**0.018 (0.006, 0.102)**	**0.015 (0.005, 0.084)**	**0.018 (0.005, 0.130)**	**0.019 (0.005, 0.097)**	**0.143 (0.065, 0.281)**
Testosterone	0.007 (0.002, 0.083)	0.007 (0.002, 0.069)	0.009 (0.001, 0.097)	0.008 (0.002, 0.097)	**0.085 (0.032, 0.226)**

Notes

All estimates are given with 95% credible intervals (i.e., highest posterior density intervals [HPDI]). Significant estimates are in bold.

a Risk‐taking was fit with the "categorical" family distribution; all other variables were fit with a Gaussian distribution. Data from both parents and pups were included in the model (see Supporting information Appendix S1: Table S2 and Table S3 for model specifications).

### 3.4 Correlation estimates

**Figure 5:**
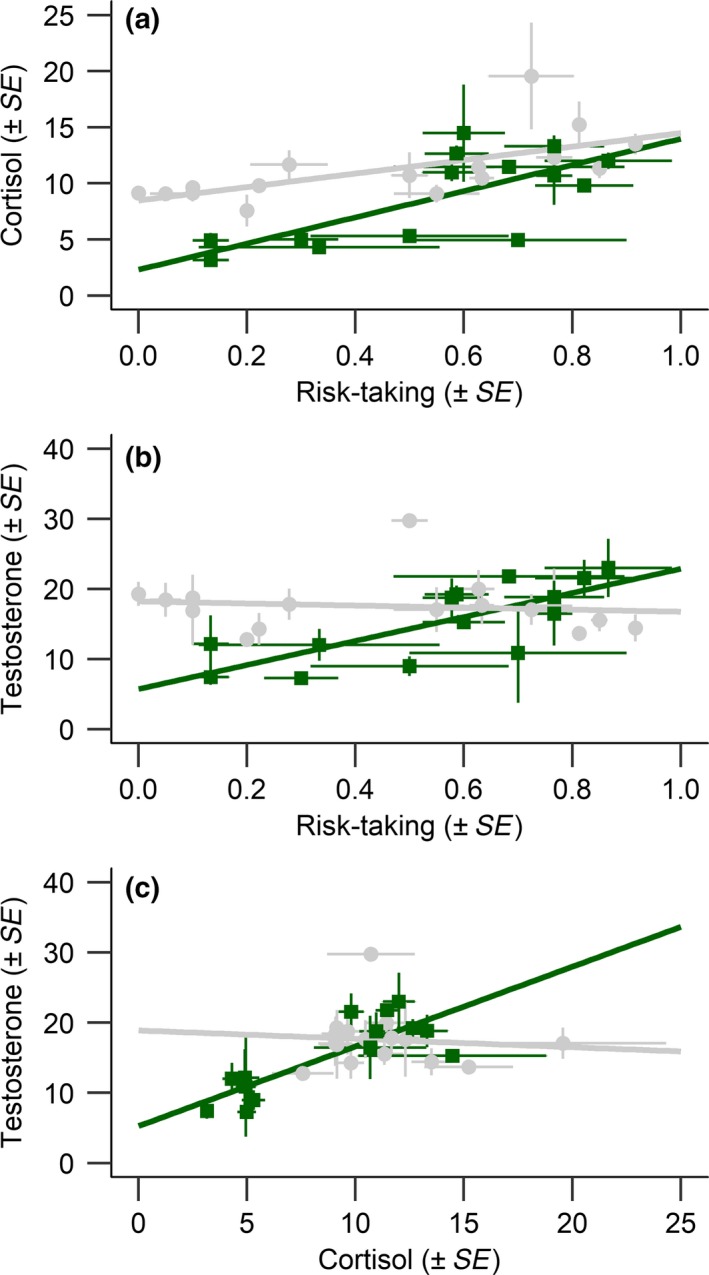
Relationships among risk‐taking and cortisol (a), risk‐taking and testosterone (b) and cortisol and testosterone (c) of offspring during the weaning (5–10 weeks of age; gray circles) and juvenile (10–15 weeks of age; green squares) stages of development. Each point represents the litter average ± *SE*

Within the weaning stage of development (i.e., 5–10 weeks of age), we found evidence of a positive phenotypic correlation among risk‐taking behavior and cortisol (Figure 5a). That correlation was strongly underpinned by substantial cohort correlations (Table 4). We did not find evidence of genetic or maternal correlations among risk‐taking and cortisol. Furthermore, we did not find support for correlations between risk‐taking and testosterone, nor between cortisol and testosterone, during the weaning stage (Table 4). Within the juvenile stage, we found evidence of positive cohort correlations for all trait combinations, and positive phenotypic correlations for two‐thirds of the trait combinations (Table 4, Figure 5). We did not find genetic or maternal correlations among traits within the juvenile stage (Table 4).

**Table 4:** Genetic, maternal, cohort, and phenotypic correlations between each pair of pup traits within the weaning (5–10 weeks) and juvenile (10–15 weeks) stages of development

Development Stage	Trait 1	Trait 2	Genetic	Maternal	Cohort	Phenotypic
Weaning (5–10 weeks)	Risk‐taking	Cortisol	0.507 (−0.321, 0.815)	0.242 (−0.705, 0.839)	**0.831 (0.285, 0.969)**	**0.362 (0.065, 0.593)**
	Risk‐taking	Testosterone	−0.302 (−0.743, 0.569)	−0.298 (−0.802, 0.780)	−0.810 (−0.943, 0.656)	−0.051 (−0.261, 0.204)
	Cortisol	Testosterone	−0.954 (−0.984, 0.895)	0.016 (−0.877, 0.783)	−0.795 (−0.958, 0.680)	0.197 (−0.105, 0.452)
Juvenile (10–15 weeks)	Risk‐taking	Cortisol	−0.095 (−0.744, 0.787)	0.020 (−0.738, 0.829)	**0.857 (0.384, 0.972)**	0.304 (−0.032, 0.599)
	Risk‐taking	Testosterone	0.493 (−0.573, 0.899)	0.283 (−0.662, 0.906)	**0.879 (0.368, 0.970)**	**0.439 (0.097, 0.651)**
	Cortisol	Testosterone	0.941 (−0.899, 0.978)	0.027 (−0.746, 0.937)	**0.969 (0.784, 0.996)**	**0.553 (0.212, 0.749)**

Note

Estimates are given with 95% credible intervals, and significant estimates are in bold.

## 4 DISCUSSION

The combination of prior experiences paired with current environmental context induce parental plasticity over multiple reproductive bouts (Plaistow et al., 2007; Uller, 2008; Uller et al., 2013), emphasizing the central role of environmental experience in driving transgenerational plasticity (Crean, Dwyer, & Marshall, 2013; Marshall, 2008; Uller, 2008). In anthropogenic contexts, parental habituation of humans may operate as a cue for offspring to modify their fear responses of humans. Our data support this hypothesis: parents were less fearful of human disturbance with their second litters, and pups from second‐litter cohorts were also more tolerant of humans than their first‐litter siblings (Figure 3, Table 1). In addition, we found evidence of parental effects on risk‐taking behavior (Table 3), which provides additional support for the role of parental identity in shaping patterns of offspring fear (Table 3). Finally, pup risk‐taking increased over development (Table 1b), suggesting that individual‐level risk is plastic and can be adjusted over ontogeny. It is well‐known that wildlife with accrued experiences of human disturbance over time become increasingly habituated to, and tolerant of, humans (Carrete & Tella, 2017; Carrete et al., 2016; Greggor, Clayton, Fulford, & Thornton, 2016; Perals, Griffin, Bartomeus, & Sol, 2017; Samia et al., 2015; Sol et al., 2013, 2018; Vincze et al., 2016). Moreover, prior work in coyotes has demonstrated that personality differences in risk can be successfully quantified via response to humans (Darrow & Shivik, 2009; Dawson & Jaeger, 2009; Gilbert‐Norton, Leaver, & Shivik, 2009; Murray, Edwards, Abercrombie, & St. Clair, 2015; Poessel, Gese, & Young, 2017; Schmidt & Timm, 2007; Young, Mahe, & Breck, 2015). The mechanisms that contribute to rapid plasticity in wildlife fear are less well‐understood (Carrete & Tella, 2017). Our results posit that one potential mechanism shaping organismal fear of humans in coyotes may be parental effects.

We provide evidence to suggest that second‐litter pups had lower testosterone at 5 weeks of age (Figure 4, Table 2), supporting our a priori prediction that offspring testosterone levels would match decreased prepartum testosterone of experienced parents found in our previous study (Schell et al., 2016). However, that trend was reversed over time, as second‐litter pups demonstrated higher cortisol and testosterone concentrations at 15 weeks of age compared to their first‐litter siblings (Figure 4, Table 2). One explanation for this trend is that the contribution of parental input to offspring endocrine traits varies over development. Infant coyotes have considerably more contact with their parents early in development (Gese, Roberts, & Knowlton, 2016). By ~6–7 weeks of age, littermates establish relatively stable social hierarchies and are more independent from their parents (Fentress et al., 1987; Kitchen & Knowlton, 2006). Thus, it may be that social interactions amongst littermates contributed more substantially to affecting offspring hormonal development than parental identity. Previous studies on vertebrate sibling competition in relation to maternal influence provide partial insight into this explanation (Carere, Drent, Koolhaas, & Groothuis, 2005; Golla, Hofer, & East Marion, 1999; Hudson & Trillmich, 2007; Wahaj & Holekamp, 2006), although none have explored how the endocrine outcomes of offspring vary over development. Alternatively, increased cortisol and testosterone of second‐litter offspring may be a function of litter size: more siblings may lead to more competition, and thus, higher stress and reproductive physiology. Litter size positively covaried with reproductive bout (Schell et al., 2018); yet, we did not find any evidence that litter size was a significant predictor of endocrine traits over development (Table 2). Another alternative explanation may simply be that individual personalities, and not sheer number of siblings, drive social dynamics that influence individual endocrine function. There is evidence in other taxa suggesting that individual‐level behavioral consistency is more salient to group function than the number of individuals in a social group (Galhardo, Vitorino, & Oliveira, 2012; Laskowski & Bell, 2014; Montiglio, Ferrari, & Reale, 2013). Altogether, these data emphasize the importance of assessing several sources of variance (e.g., maternal, common environment) at different developmental timepoints to fully understand the contribution of parental effects to offspring endocrine development.

Evaluating the genetic and environmental sources of variance in personality and endocrine function is integral to understanding the importance of such effects on evolution (Dingemanse & Araya‐Ajoy, 2015; Dochtermann & Roff, 2010; Petelle et al., 2015). We found evidence of repeatable differences in coyote risk‐taking behavior, with significant maternal and cohort effects contributing to our repeatability estimates (Table 3). These results underscore the importance of common environmental effects and maternal influence in shaping the development of risk sensitivity in coyotes. In contrast with several recent studies (Carrete & Tella, 2017; Carrete et al., 2016; Ducatez, Audet, Rodriguez, Kayello, & Lefebvre, 2017; Sol et al., 2018), we did not find evidence of heritable variation in coyote risk‐taking. This may partially be due to pedigree depth in this study (Supporting information Appendix S1: Table S1), which is an order of magnitude smaller than prior work (Carrete et al., 2016). An alternative explanation may be that personality differences in risk‐taking and fear are both contextually and developmentally plastic in this species. According to the pace of life syndrome (POLS) hypothesis, a slow‐lived species like coyotes should be more risk‐averse with infrequent human disturbance, primarily because their life history strategy largely depends on ensuring a long reproductive lifespan (Careau, Réale, Humphries, & Thomas, 2010; Hall et al., 2015). However, if slow‐lived species manage to survive and reproduce, then such strategies (i.e., risk) can readily be adjusted with accrued experiences (Sol et al., 2018). Indeed, recent work suggests that constant exposure to anthropogenic contexts can lead to divergence in behavioral strategies used to cope with human frequentation (Charmantier, Demeyrier, Lambrechts, Perret, & Grégoire, 2017; Samia et al., 2015). Hence, prior evidence citing reduced fear to people as a necessary component of rapid evolution in anthropogenic contexts infers such processes could also occur in wild and captive coyote populations (Bókony, Kulcsár, Tóth, & Liker, 2012; Carrete & Tella, 2017; Greenberg & Holekamp, 2017; Miranda et al., 2013; Vincze et al., 2016).

Our results provide evidence for the emergence of personality and phenotypic syndromes in early life history, as similarly shown in other mammals (European roe deer, *Capreolus capreolus*, Debeffe et al., 2015; dwarf hamsters, *Phodopus sungorus*, Kanda, Louon, & Straley, 2012), fish (convict cichlids, *Amatitlania siquia*, Mazué, Dechaume‐Moncharmont, & Godin, 2015), and reptiles (red‐eared slider turtles, *Trachemys scripta*, Carter, Paitz, McGhee, & Bowden, 2016). Phenotypic correlations among traits were substantially influenced by cohort identity, which suggest that the emergence of behavior‐endocrine syndromes in coyote pups is attributed to litter identity (Table 4). Correlated phenotypic suites of behavioral and endocrine traits are important because they often covary with ecological conditions, and thus are sensitive to correlational selection (Bell, 2007; Dingemanse & Réale, 2005; Miles, Sinervo, Hazard, Svensson, & Costa, 2007). Moreover, covariation among personalities and endocrine response underscore the mechanistic and functional links between the two (Boulton et al., 2015; Carere, Caramaschi, & Fawcett, 2010; Miranda, 2017; Taff & Vitousek, 2016). Our study adds to a nascent but growing anthology suggesting that personality differences in fear toward humans is mediated by endocrine function (Bonier, 2012; Rebolo‐Ifran et al., 2015). In addition, our results support evidence from previous studies suggesting that variation in the social environment during development can affect individual phenotype in social organisms (Blumstein, Fuong, & Palmer, 2017; McCowan, Mainwaring, Prior, & Griffith, 2015; Montiglio et al., 2013).

The expression of personality traits can occasionally differ amongst wild and captive individuals (Mason et al., 2013; Niemelä & Dingemanse, 2014), although recent evidence suggests personality in captivity reflects personality in wild settings (Fisher, James, Rodríguez‐Muñoz, & Tregenza, 2015; Herborn, Heidinger, Alexander, & Arnold, 2014). Captivity is generally safer with positive experiences of humans (e.g., via enrichment), whereas even in urban or protected natural settings, the potential for lethal removal still exists (Clinchy et al., 2016; Smith et al., 2017). As a result, the observed levels of risk‐taking in our population may be more exaggerated compared with that of conspecifics in urban or protected natural areas. An individual coyote's perception of risk within the captive environment may also contribute to differences in risk‐taking amongst wild and captive populations. Recent work in other species has shown that urban organisms can identify individual humans (Levey et al., 2009) and exhibit behavioral plasticity when humans diverge from a predictable behavioral pattern (Bateman & Fleming, 2014). Anecdotal evidence suggests coyotes similarly learn to identify individual humans (C. J. Schell pers. obs.), and modify their behavior according to variance in human activity (Schultz & Young, 2018; Séquin, Jaeger, Brussard, & Barrett, 2003; Smith et al., 2018). We may therefore predict that the specific person administering the foraging assay paired with unpredictable human behavior should induce plasticity in risk‐taking. Future work should compare risk‐taking both in wild and captive coyote systems, across a gradient of threat, to help elucidate how variation in anthropogenic disturbance regimes contribute to variance in habituation rates over time.

To conclude, the relationship among parent and offspring can dictate how future generations will navigate predicted ecological conditions (Badyaev & Uller, 2009; Duckworth et al., 2015; Wolf et al., 1998). The predictability of environmental cues and the transmission of those cues to offspring are particularly intriguing in an anthropogenic context, in which parents modify their phenotype according to human influence. As a result, we may expect that parents with accumulating anthropogenic experiences over multiple reproductive bouts may produce offspring that have an optimal phenotype suited to environments with increased human densities. Our results provide evidence to suggest that parental effects reduce fear in anthropogenic settings within as little as two generations. This is likely due to strong parental influence, in which parental habituation level is an ever‐present cue that offspring use to modify their fear responses toward humans. It remains unclear, however, whether reductions in fear of humans have fitness consequences for developing coyotes in the wild, as is the case for other taxa (De Meester et al., 2018; Hall et al., 2015). In addition, it is still uncertain whether such traits observed during development are consistent across multiple life stages. Future work addressing the stability of pup traits across life stages is critical to determining the importance of early developmental experiences and parental effects on individual fitness.

## CONFLICT OF INTEREST

The authors do not have any conflict of interest to declare.

## AUTHOR CONTRIBUTIONS

C.J.S. conceived the research, conducted field work, analyzed data, created figures, and wrote the manuscript. J.K.Y. conceived the research, conducted field work, provided comments on the manuscript, and provided funding. E.V.L. conceived the research and provided comments on the manuscript. R.M.S. conceived the research, assisted in endocrine lab work, provided comments on the manuscript and provided funding. J.M.M. conceived the research and provided comments on the manuscript.

## DATA ACCESSIBILITY

The datasets used for these analyses, as well as the R script used to perform the analyses, are deposited in the Dryad Digital Repository as https://doi.org/10.5061/dryad.pr00820.
