# Supplementary material for: Parental habituation to human disturbance over time reduces fear of humans in coyote offspring
Source: Ecol Evol. 2018 Dec 11;8(24):12965–80. doi: 10.1002/ece3.4741 (PMC6308887; doi:10.1002/ece3.4741)
Supplement: Supplementary file 1 [file ECE3-8-12965-s001.docx]

**TITLE**: Parental habituation to human disturbance over time reduces fear of humans in coyote offspring

**JOURNAL**: Ecology and Evolution

**Corresponding author**:

Christopher J. Schell

Email: [cjschell@uw.edu](mailto:cjschell@uw.edu)

**Table S1.** Summary statistics from the pedigree used in all models, extracted using the R package “pedantics” (Morrissey & Wilson, 2010)

| Number of founders | 22 |
| --- | --- |
| Number of full sibs | 293 |
| Non-zero F | 0 |
| F>0.125 | 0 |
| Mean pairwise relatedness | 0.068 |
| Pairwise relatedness ≥ 0.125 | 0.231 |
| Pairwise relatedness ≥ 0.25 | 0.133 |
| Pairwise relatedness ≥ 0.5 | 0.073 |
|  |  |
| **Maternal Summary** |  |
| No. of maternities | 93 |
| No. of maternal sibs | 93 |
| No. of maternal grandmothers | 71 |
| No. of maternal grandfathers | 71 |
| Mean maternal sibship size | 4.04 |
|  |  |
| **Paternal Summary** |  |
| No. of paternities | 93 |
| No. of paternal sibs | 93 |
| No. of paternal grandmothers | 77 |
| No. of paternal grandfathers | 77 |
| Mean paternal sibship size | 4.04 |

**Reference**

Morrissey, M. B., & Wilson, A. J. (2010). Pedantics: An r package for pedigree-based genetic simulation and pedigree manipulation, characterization and viewing. *Molecular Ecology Resources*, *10*(4), 711–719. http://doi.org/10.1111/j.1755-0998.2009.02817.x

**Fig. S1** Risk-taking behavior (i.e., foraging rate) during the first- and second breeding seasons of each parent-pup family unit (a-h), and the average risk-taking of all mothers, fathers, and litters (i). Lines for mothers (M#) and fathers (D#) denote individuals over time; whereas lines for litters compare first- and second-litter siblings.

Reproductive bout

**Table S2**. Model selection analysis for risk-taking behavior comparing the null model with all fixed effect terms (i.e. developmental age, age class, litter size, sex, prepartum odor treatment, and litter year), and an alternative model with an additional interaction term between Litter size and Litter Year. Data for subsequent model comparisons were partitioned into three categories: (a) all age classes, (b) within the pup age class, and (c) within the adult age class. Deviance information criterion (DIC) and delta DIC were used to assess model fit. All models included the random terms 'animal', 'ID', 'dam', and 'litter identity'. Estimates under each fixed effect are the mode of the posterior distribution (ß), and a ‘+’ indicates that values are approximately identical between compared models.

| **(A) all age classes** | **(Intercept)** | **Age** | **Age class** | **Litter Size** | **Sex** | **Odor** | **Litter Year** | **Size*Year** | **df** | **DIC** | **ΔDIC** |
| --- | --- | --- | --- | --- | --- | --- | --- | --- | --- | --- | --- |
| Model 1.1 | -2.38 | 0.10 | + | 0.51 | + | + | + |  | 12.00 | 2329.50 | 0.00 |
| Model 1.2 | -3.08 | 0.10 | + | 0.72 | + | + | + | + | 13.00 | 2329.60 | 0.10 |
|  |  |  |  |  |  |  |  |  |  |  |  |
| **(B) within pups** | **(Intercept)** | **Age** | **Age class** | **Litter Size** | **Sex** | **Odor** | **Litter Year** | **Size*Year** | **df** | **DIC** | **ΔDIC** |
| Model 2.1 | -3.10 | 0.11 |  | 0.07 | + | + | + |  | 11.00 | 1879.70 | 0.00 |
| Model 2.2 | -2.77 | 0.11 |  | -0.03 | + | + | + | + | 12.00 | 1879.70 | 0.00 |
|  |  |  |  |  |  |  |  |  |  |  |  |
| **(C) within adults** | **(Intercept)** | **Age** | **Age class** | **Litter Size** | **Sex** | **Odor** | **Litter Year** | **Size*Year** | **df** | **DIC** | **ΔDIC** |
| Model 3.1 | -2.20 | 0.06 |  | 0.73 | + | + | + |  | 11.00 | 442.10 | 0.00 |
| Model 3.2 | -3.37 | 0.06 |  | 1.07 | + | + | + | + | 12.00 | 442.70 | 0.65 |

**Table S3**. Model selection analysis comparing the null model with all fixed effect terms (i.e. developmental age, age class, litter size, sex, prepartum odor treatment, and litter year), and first alternative model with an additional interaction term between Litter size and Litter Year, a second alternative model with an interaction term between developmental age and litter year, and a third alternative model with all possible interactions among Litter Year, Age, and Litter Size. Deviance information criterion (DIC) and delta DIC were used to assess model fit. All models had the random terms of 'animal', 'ID', 'dam', and 'litter identity'. The best-fit model is in bold.

| (A) Cortisol | (Intercept) | Age | Litter Size | Sex | Odor | Litter Year | Age*Year | Size*Year | Age*Size | Age*Size*Year | df | DIC | ΔDIC |
| --- | --- | --- | --- | --- | --- | --- | --- | --- | --- | --- | --- | --- | --- |
| Model 1.1 | 11.44 | -1.96 | 0.55 | + | + | + |  |  |  |  | 11.00 | 1117.20 | 32.80 |
| Model 1.2 | 11.17 | -1.95 | 0.63 | + | + | + |  | + |  |  | 12.00 | 1118.60 | 34.20 |
| **Model 1.3** | **15.91** | **-4.38** | **0.54** | **+** | **+** | **+** | **+** |  |  |  | **12.00** | **1084.40** | **0.00** |
| Model 1.4 | 13.92 | -3.39 | 1.04 | + | + | + | + | + | -0.25 | + | 15.00 | 1089.00 | 4.60 |
|  |  |  |  |  |  |  |  |  |  |  |  |  |  |
| (B) Testosterone | (Intercept) | Age | Litter Size | Sex | Odor | Litter Year | Age*Year | Size*Year | Age*Size | Age*Size*Year | df | DIC | ΔDIC |
| Model 4.1 | 9.24 | 3.27 | -0.18 | + | + | + |  |  |  |  | 11.00 | 1344.30 | 34.81 |
| Model 4.2 | 11.09 | 3.24 | -0.70 | + | + | + |  | + |  |  | 12.00 | 1345.30 | 35.83 |
| **Model 4.3** | **17.65** | **-1.23** | **-0.23** | **+** | **+** | **+** | **+** |  |  |  | **12.00** | **1309.50** | **0.00** |
| Model 4.4 | 21.68 | -2.12 | -1.29 | + | + | + | + | + | 0.22 | + | 15.00 | 1314.10 | 4.63 |

**Table S4.** Heritability (h2), maternal effects (m2), permanent environmental effects (PE), cohort effects (C), and repeatability (r) of coyote risk-taking behavior within each age class. Estimates are given with 95% credible intervals, and significant estimates are in bold.

| **Age Class** | ***h^2^*** | ***PE*** | ***m2*** | ***C*** | ***r*** |
| --- | --- | --- | --- | --- | --- |
| Pup | 0.001 (0.000, 0.171) | 0.001 (0.000, 0.124) | 0.001 (0.000, 0.166) | **0.097 (0.000, 0.256)** | **0.257 (0.148, 0.441)** |
| Adult | 0.002 (0.000, 0.410) | 0.001 (0.000, 0.411) | 0.001 (0.000, 0.361) | **0.131 (0.012, 0.379)** | **0.601 (0.410, 0.739)** |
